# Sensors Based on Bio and Biomimetic Receptors in Medical Diagnostic, Environment, and Food Analysis

**DOI:** 10.3390/bios8020035

**Published:** 2018-04-01

**Authors:** Alisa N. Kozitsina, Tatiana S. Svalova, Natalia N. Malysheva, Andrei V. Okhokhonin, Marina B. Vidrevich, Khiena Z. Brainina

**Affiliations:** 1 Department of Analytical Chemistry, Institute of Chemical Engineering, Ural Federal University named after the first President of Russia B.N. Yeltsin, 620002 Yekaterinburg, Russia; a.n.kozitsina@urfu.ru (A.N.K.); tanyamitrofanova1990@mail.ru (T.S.S.); n.n.malysheva@urfu.ru (N.N.M.); a.v.ohohonin@urfu.ru (A.V.O.); 2 Scientific and Innovation Center for Sensory Technologies, Ural State University of Economics, 620144 Yekaterinburg, Russia; mbv@usue.ru

**Keywords:** biosensor, mimetic receptor, non-enzymatic sensor, aptamer, molecular imprinted polymer, transducer, electrochemical sensors, wearable and written sensors, self-powered sensors

## Abstract

Analytical chemistry is now developing mainly in two areas: automation and the creation of complexes that allow, on the one hand, for simultaneously analyzing a large number of samples without the participation of an operator, and on the other, the development of portable miniature devices for personalized medicine and the monitoring of a human habitat. The sensor devices, the great majority of which are biosensors and chemical sensors, perform the role of the latter. That last line is considered in the proposed review. Attention is paid to transducers, receptors, techniques of immobilization of the receptor layer on the transducer surface, processes of signal generation and detection, and methods for increasing sensitivity and accuracy. The features of sensors based on synthetic receptors and additional components (aptamers, molecular imprinted polymers, biomimetics) are discussed. Examples of bio- and chemical sensors’ application are given. Miniaturization paths, new power supply means, and wearable and printed sensors are described. Progress in this area opens a revolutionary era in the development of methods of on-site and in-situ monitoring, that is, paving the way from the “test-tube to the smartphone”.

## 1. Sensors Characteristics

In analytical chemistry, two main types of sensors are usually considered: biological and chemical ones. Generally, biosensors/sensors are classified according to the type of the receptor:

- enzymes,
- affinity receptors (antigens/antibodies, DNA probes),
- cells and cell organelles,
- artificial receptors (Molecularly Imprinted Polymers, biomimetics, aptamers).

The sensors also differ by the method for immobilization of the receptor layer (physical sorption, immobilization in a polymer matrix, covalent binding, affinity immobilization) and method for an analytical signal’s detection (optical, electrochemical, fluorescent, etc.).

Any sensor contains a transducer that changes one form of energy into another. In an electrochemical sensor, an electrode plays the role of a transducer. Another important part of the sensor is the receptor-sensitive recognition element. The main difference between biological and chemical sensors is the nature of a receptor. In the first case it is a biomaterial, and in the second case it is a chemical compound or a group of compounds selectively interacting with an analyte.

A variety of biological and synthetic materials and ways of their immobilization on the transducer and the use of nanotechnologies lead to the appearance of a steadily increasing number of biosensors/sensors.

### 1.1. Thin- and Thick-Film Transducers

In electrochemical sensors, electrodes perform the role of transducers. Requirements for working electrodes are high electroconductivity, inertness over a wide potential range, non-toxicity, and, finally, low cost. The rejection of an extremely toxic mercury electrode opened the era of solid-phase sensors [1,2,3]. In the development of sensors today, as a rule, planar electrodes and electrode systems made by screen printing (thick-film screen-printed electrodes) or thin-layer deposition are used [4,5]. As an alternative to mercury, low-toxicity solid-phase compounds [6,7], noble metals [8,9], carbon [10], or composite (nano)materials [11,12] are used.

Planar electrodes, based on noble metals, are made by thin-layer sputtering. Photolithography, vacuum deposition, etching, and stepwise firing are used to obtain thin metal films of gold, silver, platinum, etc. on the surface of the substrate [13,14].

Electrodes based on carbon paste are prepared by mixing carbon powder with paraffin, petroleum oils, dioctylphthalate, α-bromnaphthalene, or tricresyl phosphate (“carbon paste electrodes”) and by a screen-printing process. Pastes for electrode printing consist of fine carbon material and epoxy resins, etc. [15,16]. The non-working area of the transducer is insulated using a dielectric. This technology makes it possible to obtain thick films (about 20 μm).

Thus, the main advantages of thick-film electrodes are their low cost, simplicity of manufacturing, compactness, and providing a wide choice of materials and designs [17]. In addition, it is not necessary to perform the procedure for cleaning and preparing the electrode surface before each measurement, since the electrode is intended for one-time use. The disadvantages include the inability to analyze organic media capable of dissolving paste components in the conductive layer. However, examples of the use of organic-solvent-compatible screen-printed electrodes have been published [18].

Planar electrodes have found a wide application in easy-to-use portable point-of-care sensors, which can be performed at the bedside and in a “home clinic”.

### 1.2. Receptors

The main focus in the development of receptors is currently on enzyme and affinity biosensors.

*Enzyme biosensors*. In this case, the receptor properties are determined by the ability of natural enzymes to participate in ultraspecific catalytic reactions with certain substrates–analytes [19,20]. Enzymes are protein macromolecules that have a substrate-binding site and an active site providing a catalytic reaction. Some enzymes have in their structure a cofactor, which is a non-protein component responsible for catalysis (metal ions, iron-sulfur clusters, flavins, gems, etc.). The enzyme activity strongly depends on environmental conditions, such as temperature, ionic strength and pH of the solution, and the presence/absence of inhibitors. [21,22]. The disadvantages of enzyme sensors are determined by their low stability, short shelf life, and high cost.

Tissue and cells in some cases serve as receptors in biosensors, while the receptor functions perform the reactions with enzymes contained in them [23,24,25,26]. The simplest microorganisms, such as bacteria, yeast, and fungi, and their separate organelles, for example, cell membranes, are the most frequently used as recognition elements in cell biosensors. Tissue biosensors contain homogenates or thin sections of plant and animal tissues immobilized on the surface of the transducer. Such receptors do not require the use of expensive procedures for the isolation and purification of enzymes. Furthermore, in them enzymes are more stable than those in pure form due to the “habitual” environment [25,27,28,29,30].

In *affinity biosensors*, reversible biochemical processes for the formation of complementary complexes are used. DNA sensors and immunosensors relate to this group. In the first case, the receptor is a DNA fragment. Examples are DNA probes or DNA primers [31,32]. In an immunoassay, an antibody or an antigen serves as a receptor. The formation of an immunocomplex is provided by a Fab fragment of the antibody. Variable and hypervariable regions in their structure are energetically and conformationally complementary to the antigen [33,34]. The library of antibodies and DNA probes is enormous today, and the processes for obtaining, isolating, and purifying such receptors are automated. In addition, DNA primers and antibodies are more stable and universal than enzymes.

In *chemical sensors*, receptors are synthetic molecules having in their structure functional groups able to selectively interact with an analyte. They are used in sensors for the determination of both individual chemical compounds and groups of substances, which are implemented in chemometric systems, such as an “electronic nose” or an “electronic tongue” [35,36].

This kind of sensor for bioanalytes determination contains the following types of compounds as receptors:

(1) non-biological molecules modeling the active sites of an enzyme–redox-active metal centers and suitable ligand environment that provide electrocatalytic processes simulating the action of enzymes [37],
(2) molecularly imprinted polymers [38,39],
(3) calixarenes [40,41], and
(4) nanomaterials and others [42].

Synthetic receptors are more stable than biological ones, have a relatively small molecular weight, and bind more firmly to the analyte. Their main disadvantage is their relatively low biocompatibility.

### 1.3. Methods of Immobilization

To improve the analytical characteristics of sensors and expand the range of analytes, the surface of the transducer is modified with organic compounds, nanomaterials, enzymes, affinity agents [43,44,45], etc. A modifier is either “grafted” onto the surface (surface modification) or mixed with components of electrode pastes (bulk modification).

The basic requirements for immobilization methods are maximum preservation of specificity and exclusion of receptor degradation (the activity of a recognition component in its immobilized state should be comparable with its activity in a solution). The processes for immobilization of a receptor layer in chemical sensors and biosensors are based on general principles. However, in the case of biosensors it is especially important to perform the procedure in the most “soft”, close to physiological, conditions [46,47]. The main methods for immobilization of the receptor are physical sorption, incorporation into a polymer matrix, covalent fixation, and immobilization using affinity reagents.

*Physical sorption* is the simplest way to immobilize a receptor based on its electrostatic and Van der Waals interaction with the transducer material [48,49]. This method is widely used due to the simplicity of implementation and low cost. However, the low stability of these sensors during storage and the inability to re-use them and regenerate their surface are essential disadvantages of this type of immobilization [50].

*Immobilization of a bioreceptor into a polymer matrix* is most often performed by photopolymerization [51], electrochemical polymerization, or incorporation into a sol-gel matrix [52]. Polymers are increasingly frequently being further modified with various functional groups (-COOH, -NH_2_, etc.) to hold the bioreceptor/receptor more firmly and orient it. Thus, the receptor located in the polymer pores, on the one hand, is reliably retained on the transducer’s surface, and on the other hand, its reactivity is preserved. This method, as well as physical sorption, is most often used for the immobilization of enzymes [53,54] and living cells [55]. In addition, the method is simple and it significantly prolongs the receptor’s activity. However, polymerization requires strict precision in the experimental conditions, specific pore size, the polymer’s composition, and the sorption-desorption equilibrium of the system.

*The method of covalent immobilization* consists in modifying the transducer’s surface by compounds capable of forming chemical (in particular, covalent) bonds with components of the receptor layer. Cross-linked reagents–thiols [56], amines [57], carboxyl compounds [58], alkynes, azides [59], and so on are most frequently used as modifiers. This method provides a firm and oriented fixing of the receptor on the transducer’s surface. It promotes a significant increase in the sensitivity and accuracy of detection, and allows for surface regeneration, for example, by changing the stability of the “analyte-receptor” complex [60,61]. This increases the shelf life of the sensor. However, the use of toxic reagents and extreme environmental conditions (high/low pH values, a non-physiological temperature, and organic solvents) often does not allow for using this method for the immobilization of biomaterial.

*Affinity immobilization*. In this case, the layer applied on the transducer’s surface includes reagents capable of interacting with the bioreceptor, thereby fixing the bioreceptor on the surface. Complementary sites of DNA [62], genetically modified polypeptides [63], and individual amino acids and aptamers [64] act as such agents. The method is universal and highly specific; it does not lead to degradation of the bioreceptor, but it is labor-consuming. In addition, massive protein molecules on the transducer’s surface negatively affect the sensitivity of the analysis, for example, in the case of electrochemical sensors.

Despite the wide choice of existing methods for immobilization, the listed problems cannot be considered solved. The search for and synthesis of new linkers that are non-toxic and biocompatible with the living cell and/or its individual organelles as well as improving immobilization techniques are highly relevant.

The use of synthetic receptors opens new possibilities for their immobilization, mainly by expanding the range of potential linkers and by the high tolerance of such molecules to aggressive environmental conditions.

### 1.4. Signal-Forming Processes

**Figure 1:**
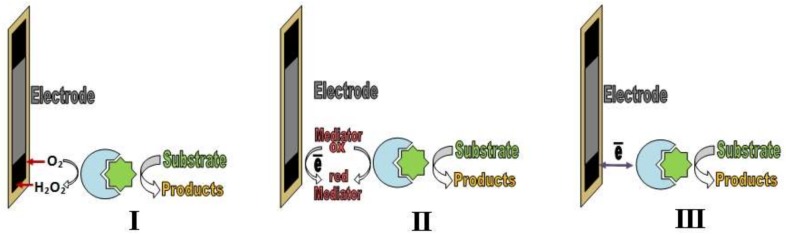
First (I), second (II) and third (III) generations of enzyme sensors

The signal-forming process in enzyme sensors, as a rule, includes one or several catalytic transformations of a substrate (analyte) [65]. Several generations of enzyme sensors exist (Figure 1). In the *first generation* of enzyme sensors (I), the product of a catalytic interaction enters into an electrochemical signal-forming reaction. In the *second generation*, the signal-forming process includes the interaction of the product of the enzymatic reaction with a mediator (II), and an analytical signal forms because of the exchange of electrons between the mediator and a transducer. In the *third generation* (III) of enzyme sensors, direct electron transfer between the transducer and the active center of the enzyme determines the analytical signal [20].

DNA hybridization and DNA intercalation sensors are described as follows. DNA *hybridization* sensors are based on the formation of double-stranded DNA between a DNA probe and an analyte of a sample. The basic principle of *intercalation* devices is the ability of the analyte to embed into the structure of a DNA probe.

Immunoassay methods are based on the detection of either an antigen–antibody complex (Ag–Ab) or excessive amounts of immunoreagents (antigens or antibodies) [62]. Unlike enzyme sensors, the components of affinity complexes are not able to generate a distinct and reproducible analytical signal.

In this case, an enzyme is used as a label, which takes part in the formation of the response. As a rule, antigens, antibodies, and DNA primers are labeled. Homogenous (formation of an immunocomplex antigen–antibody in solution) and heterogeneous (formation of an immunocomplex using a solid template) methods are known for enzyme immunoassays.

The main disadvantage of an enzyme immunoassay is the labor-consuming and multi-stage analysis. For example, classical ELISA includes, in addition to the formation of the “antigen–antibody” immunocomplex, the subsequent localization of an enzyme-labeled conjugate of anti-antibodies on an immunocomplex. It is possible to reduce the number of stages by implementing a competitive and non-competitive format for heterogeneous analysis immunoassays using signal-forming label (able to generate an analytical signal by itself) and label-free procedures [66].

### 1.5. Methods of Analytical Signal Detection

Methods of analytical signal detection depend on the physicochemical properties of the receptor, and its ability to generate one or the other type of signal correlates with analyte concentration. The type of the signal and available detector determines the measurement method. There are many phenomena, effects, and types of energy converters that can be used in sensor designs. Detectors based on changes in thermal conductivity [67], the appearance/suppression of thermal radiation [68,69,70], the appearance of an electromotive force with rising temperature [71], Faraday [72] and Doppler [73] effects, the piezoelectric effect [74,75], etc. are known. The most common are optical and electrochemical detectors.

In optical sensors, the signal-forming process involves a change in phase, amplitude, polarization, and frequency of the light because of the “analyte–receptor” complex’s formation. Signal-forming labels (colorimetric and luminescent) and label-free ones (based on surface plasmon resonance (SPR), combinatorial light scattering, etc.) are used in optical sensors.

Colorimetric sensors are available and easy to use, but are suitable for qualitative/semi-quantitative analysis and much less sensitive than, for example, luminescent sensors. Photo-/fluo-/bio-/chemiuminescences can be distinguished and have become popular [76]. Moreover, in many sensors, a strategy of amplified detection, for example electrochemiluminescence, is used. Оrganic dyes [77,78,79] and quantum dots [80,81,82,83,84] are applied as signal-forming labels in optical affinity biosensors. Due to the high sensitivity and wide range of detectable analytes, the sensors of this kind have become popular. However, the main problem is the nonspecific luminescence, which limits the use of luminescent sensors in the analysis of real samples.

Optical sensors have several advantages: (i) each type of analyte can be determined using the appropriate spectrometric method; (ii) they create the possibility for remote monitoring; and (iii) they can be used in the implementation of non-invasive formats of biosensors [85,86]. However, some disadvantages complicate their development. These are: interference with ambient light; possible bleaching of dyes and other auxiliary components; a high signal of a background; a rather long measurement time; and a limited accessibility of accessories.

SPR sensors are a relatively new class of devices based on the reflection of incident light followed by the activation of “surface plasmons” on the metal–dielectric interface. An analytical signal is a shift of the SPR’s minimum due to affinity complex formation. This shift is greater for the more specific interactions that occur on the sensor [87,88,89,90,91]. The enormous increase in the efficiency of light scattering by molecules adsorbed on the surface of silver, gold, or copper—the phenomenon of combinatorial light scattering (CLS)—has also found a use in the development of sensors based on the interaction of a receptor and an analyte [92,93,94,95]. SPR and CLS methods are ultrasensitive, but require strict observance of the sensor’s preparation and analysis conditions, especially when treating of the transducer’s surface. Therefore, the prospects for the use of SPR and CLS sensors are not very wide.

Great attention has been paid to biosensors/sensors based on electrochemical methods of signal recording (about 80% of publications according to Scopus). Several types of *electrochemical sensors* are described in monographs [96,97], reviews [98], and original papers [99,100,101].

Voltammetric and amperometric sensors have been developed [102,103,104,105,106,107]. Amperometric sensors have found the widest application as they are the most sensitive, selective, and simple measuring devices. Voltammetric measurements include linear (LSV), pulse (DPPV), and square wave (SWV) [108,109,110,111,112,113] modes. Better selectivity and sensitivity of sensors are observed in DPPV and SWV in comparison with LSV. To increase the sensitivity, an additional step of “accumulation” of an analyte with subsequent detection of products of its electrochemical transformation—inverse voltammetry—can be introduced into the analysis procedure [3,114,115]. The potential range where the signal is observed is a qualitative characteristic and the limiting or peak current is quantitatively related to the concentration of an electroactive compound.

In potentiometric sensors, ion-selective electrodes are used as the indicator electrodes. Their potential serves as an analytical signal [116,117,118,119].

Conductometric and coulometric sensors are described as well [120,121,122,123,124,125].

The impedimetric method is based on measuring the working electrode’s resistance before and after affinity complex formation [126,127,128,129]. The electrochemical impedance spectra are recorded in the presence of electron-transfer mediators (potassium/ruthenium hexacyanoferrates, ferrocene) by applying a potential at which the redox system is electroactive and varies with a small amplitude. Impedimetric methods are similar to the methods for conductometric sensors, but signal processing is estimated using the different contribution of the capacitance and the resistance of a double electrical layer.

Field-effect transistors are actively used as miniature devices today. The work of such devices is based on a change of the electrical resistance of the conductive channel that changes due to the formation of specific “analyte–receptor” complexes. The electrons move from source to drain, and the control of the conductivity is regulated by the gate [130,131,132,133,134]. The analytical signal does not directly depend on the mass/dimensions of the complex but is determined by the charge redistribution at the gate of the transistor after interaction between the analyte and receptor. Because of this, field-effect transistors are more sensitive and accurate as sensors compared to impedimetric ones.

However, the chemical purity of the sensitive element and the immobilization of the receptor layer in this case represent a more serious problem due to an extremely small size of the transducer working area and the need for targeted immobilization of receptors only within these limits. In addition, the sample matrix effect is much more pronounced so surface protection is necessary.

The electrochemical methods are extremely attractive. The reasons are a wide choice of electrochemical reactions that can be used to form a signal, simple and relatively low-cost detectors, reliability, the low detection limits and small operating volumes of a sample, and the possibility to analyze colored samples. The possibility to analyze colored samples is especially important in the analysis of biological samples [135,136,137]. In addition, electrochemical methods do not assume any strict requirements for the shape or size of transducers and can be used in flowing, portable, and automated analytical systems. Therefore, the most commercially successful sensors are electrochemical devices, such as amperometric enzyme glucose meters (OneTouch, AccuCheck, Contour, etc.).

## 2. Non-Enzymatic Sensors

The development of biosensors is limited by their low stability and the rapid degradation of biological components during storage and use. This makes calibration difficult and reduces reliability. In contrast to biological components, their synthetic equivalents are stable chemically and thermally. Therefore, at present, the attention of researchers is being increasingly drawn to the creation of artificial analogues of enzymes: biomimetics. Recent research has been devoted to new strategies for creating biomimetics based on a deeper understanding of the fundamental principles of “guest-host” supramolecular systems design [138,139]. Natural enzymes have two important centers: (i) the cofactor; and (ii) the protein environment that provide both selectivity as a result of interaction between the analyte and receptor and a catalytic chemical reaction followed by the formation of an analytical signal.

In non-enzyme methods and sensors, the task of analytical signal formation is usually solved using non-protein catalysts. Providing selectivity in non-enzyme methods and sensors requires the creation of materials capable of molecular recognition. Sensors based on non-enzyme signal-forming labels or mediator systems (label-free sensors) in immuno- and DNA sensors are discussed in Section 3. About one third of all enzymes are “metal enzymes” and contain metal ions, which play an important role in catalysis [140]. One of the approaches to the synthesis of non-biological molecules is modeling an active site of an enzyme: a redox-active metal center and a suitable ligand environment. In these sites, catalytic processes simulating the action of hydrogenases and metal enzymes are possible [141]. For example, the authors of [142] developed a biomimetic for the heterogeneous catalysis of imines hydrogenation. A metal-organic framework modified with Brønsted acid was used as the basis. Encapsulated Cr^3+^ ions and sulfonic acid provide for high reactivity of the catalyst and the possibility of its re-use. Application of the developed biomimetic makes it possible to efficiently convert both aldimines and ketimines to their hydrogenated counterparts in a similar way to enzymatic catalysis.

The novel paddle-wheel Ni complex was synthesized due to the electro-oxidation of a dinickel precursor [143]. It allowed for the production of nickel (II) hydroxide nanoparticles on an electrode’s surface for the electrocatalytic detection of sugars. The small size (1–3 nm) and effective electrodeposition of the nanoparticles played key roles in the improvement of the sensitivity for sugars detection (C_lim_ = 0.4 µM for glucose). Macrocyclic complexes of Ni (II) were synthesized [144] and their catalytic activity in the oxidation of amines was studied. A chronopotentiometric method for urea and creatinine determination was developed [145]. Many papers devoted to the electrocatalytic oxidation of alcohols, vitamins, amino acids, insulin, and urea in the presence of complexes of transition metal ions have been published [146,147,148,149]. Particularly, Ni(OH)_2_ films formed on the nickel anode in a strongly alkaline medium were used for the electrocatalytic detection of alcohols and amines [150]. Complexes of zinc with 1,4,7,10 tetraazacyclododecane (cyclen) incorporated into poly (ethylene glycol) dimethylacrylate were applied to the potentiometric detection of creatinine [151]. The main advantage of metal complexes is the ability not only to catalyze the transformation of the substrate, but also to coordinate it due to the ligands near the metal center of the biomimetic.

Many works on the investigation of the catalytic activity of organic polymers without metal in their structure have been published [152,153], but their catalysis efficiency is relatively low. Therefore, organic polymers are not widely used as biomimetics.

Nanomaterials are actively used in the creation of synthetic enzymes, both as a carrier for catalyst molecules and using their own catalytic activity. Investigations in this area are described in a new review [96]. Two aspects are considered: the impact of surface Gibbs free energy on decreasing the chemical reaction barrier and catalytic effects. An interesting experimental work [154] in which the synthesis of several types of nanoparticles (gold, silver of various structure and composition, including individual metals, core–shell particles, and nanoalloys) and their application in a reaction of cholesterol oxidation has been published. A biomimetic based on Fe_3_O_4_ nanoparticles for non-enzymatic H_2_O_2_ detection was created [155]. The selectivity of the sensor is provided with a molecularly imprinted polymer (MIP), which includes cetyltrimethylammonium bromide (CTAB) and poly (sodium 4-styrenesulfonate). The detection limit of the sensor is 10^3^ µM/L. Silver nanoparticles were used in moxifloxacin hydrochloride determination [156] using a modified carbon-paste electrode. The detection limit of the sensor in human urine is 29 nM. Papers concerning the non-enzymatic detection of hydrogen peroxide and glucose on a base of carbon nanotubes and graphene catalytic activity have been published [157,158,159,160]. The large surface area of the nanoparticles makes it possible to successfully use them as a platform for the immobilization of agents for molecular recognition. High electrical conductivity contributes to an increase in the sensitivity of the sensor [135,155]. A serious difficulty in the commercialization of such sensors is their low reproducibility, especially when using nanomaterials. The main reason is the variation in the size, shape, or composition of nanoparticles and the general instability of their suspensions. Therefore, it is necessary to strictly control the structure of nanomaterials at the stage of their synthesis and when including them into the structure of sensors.

**Figure 2:**
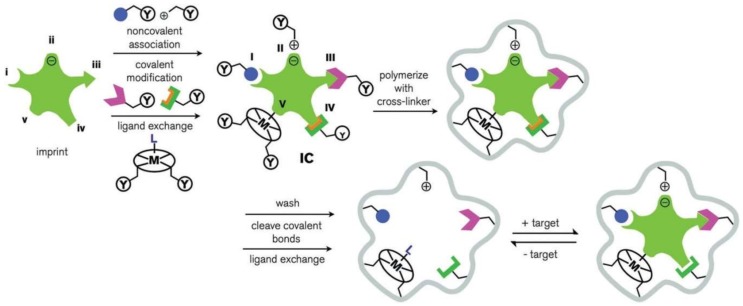
Main types of molecular imprinting [163].

An alternative to enzymes in the field of selective extraction of an analyte from a sample matrix are *molecularly imprinted polymers* (MIPs). Technologies of molecular imprinting are aimed at creating recognition sites in synthetic polymers. Inside the polymer matrix, a “template” (atoms, ions, molecules, complexes, or even microorganisms) creates cavities that correspond to the target molecule in shape, size, and energy [161,162]. In this way, the molecular imprints of the template in the polymer matrix provide exceptional selectivity for separation of the target molecules [163,164]. Organic monomers (acrylic and methacrylic acids, vinylpyridine, vinylimidazole, pyrrole, etc.) and cross-linking agents (ethylene glycol dimethacrylate, divylbenzene, etc.) can be used for the synthesis of MIPs [165,166]. The matrix can also include micro- or nanoparticles of an inorganic material (metal, SiO_2_, etc.) coated with a polymer shell [167,168,169,170,171]. The main types of molecular imprinting for the synthesis of MIPs are shown in Figure 2.

Increasing interest is currently being given to the strategy of synthesizing MIPs on the surface of nano/microparticles [172,173,174]. The use of this method allows us to obtain monodisperse particles with a high specific surface area. In addition, the application of this synthesis methodology makes it possible to use an MIP not only as a non-enzyme receptor but also as a signal-forming layer. Nanoparticles of silica [175,176], magnetite [177,178], and carbon nanotubes [179] previously modified with vinyl groups using sol-gel methods [180] and obtained by grafting [181,182] are used to implement this approach. Yet another application of MIPs is the development of new non-enzyme systems capable of catalyzing chemical reactions for which enzymes do not exist (for example, Diels–Alder reactions [183] and others) or improving the reliability, selectivity, and efficiency of already existing enzyme sensors [184].

Prospects for the development and use of MIPs are obvious, especially for the creation of simple portable devices. They will significantly improve the quality and reliability of an analysis. However, it is necessary (i) to increase the affinity to the target analyte, (ii) to effectively remove the template molecules after the synthesis of the MIP, and (iii) to exclude the non-specific adsorption of both the molecules of the analyte and those of interfering components (iv). This will allow for the wide implementation of such devices in analytical practice [185].

Selectivity of non-enzymatic sensors can be provided by *aptamers*. Aptamers are synthetic single-stranded DNA or RNA fragments of several tens of nucleic acids in length. The idea of using short sequences of nucleic acids as receptors for the selective capture of biomolecules first appeared in 1980 and continues to be developed intensively. This is due firstly to the high affinity of aptamers comparable to monoclonal antibodies (the dissociation constant in the nanomolar and picomolar range) [186]. Aptamers have in their structure clefts and grooves of target molecules, a higher receptor density, and a smaller spatial blockage that allows for increasing the binding efficiency. Secondly, aptamers are more stable in a wide range of temperatures and other extreme conditions for their storage and use in comparison with proteins [187]. Thirdly, aptamers can be synthesized chemically, which favorably distinguishes them, for example, from monoclonal antibodies, in the production of which complex and expensive technologies are used. Aptamers are universal: they can bind not only large organic ligands but also low-molecular compounds (amino acids, organic dyes, metal ions) [188,189]. Examples of practical applications of aptamers in sensory technologies are given in Section 5.

Thus, the use of supramolecular compounds, nanomaterials, electrically conductive polymers, MIPs, and aptamers as biomimetics makes non-enzymatic sensors not only a good alternative to traditional enzyme biosensors, but allows in some cases for the ability to exceed them in terms of universality, tolerance to environment, and shelf life. However, biomimetics are inferior to natural enzymes in the efficiency of their catalysis and their selectivity in extracting the substrate. Thus, the catalytic activity of metal complexes does not always correspond to enzymes, the suspension of nanomaterials is often unstable, and MIPs are not always able to provide the required affinity. There is a continuous search for new biomimetics and rational ways of obtaining them.

## 3. Methods of Sensors Characteristics Improvement

Nanomaterials are actively used as components of the modifying layer of transducers to improve the analytical characteristics of biosensors/sensors [96]. The creation of a nanomaterial layer on the transducer’s surface opens wide possibilities for the immobilization of various compounds, including biological ones. This leads to an increase in sensitivity. Recent advances in materials science and technology have made it possible to obtain nanostructured materials of various geometries that are used for conjugation with many biomolecules. The most widely used ones are quantum dots, carbon nanomaterials (nanotubes, graphene), metal nanoparticles, and hybrid core–shell nanomaterials [190,191].

*Quantum dots* are luminescent semiconductor nanocrystals that have attracted the attention of researchers due to their high quantum yield and molar extinction coefficients, wide excitation spectra, and narrow and symmetrical emission bands (30–50 nm) [192,193]. The main advantage of quantum dots is the possibility of high-precision control over their size as well as availability. The main disadvantages are their high toxicity and complexities in their synthesis [194]. In biosensors and sensors, such nanomaterials, as a rule, are used as signal-forming labels [195,196,197]. For example, a simple, fast, ultra-sensitive electrochemiluminescent competitive assay has been developed [198], where the conjugate of antibodies with CdSe acted as a signal-forming label. Gold nanoparticles were used as a carrier. Gold nanoparticles effectively accelerate the electron transfer to the transducer. The detection limit is 0.0084 ng/cm^3^ for clenbuterol detection.

Carbon nanomaterials (graphene and its oxides, fullerenes, single- and multi-walled carbon nanotubes, nanowires, fibers, sheets, etc.) are widely represented in sensors as the main material and/or a transducer modifier [199,200,201]. Currently, many reviews [39,202,203] and original papers [204,205,206] on various aspects of carbon nanomaterials application in sensors and biosensors have been published. These materials have a developed surface and high conductivity and mechanical strength. The morphology of carbon materials promotes an increase in the sensitivity, selectivity, and stability of the response compared with an unmodified transducer [207]. In the work [208], multi-walled carbon nanotubes have been successfully used as an electrically conductive layer of a developed DNA sensor for determination of hepatitis B.

The main disadvantages observed in using such materials are hydrophobicity and the tendency to aggregate.

*Nanoparticles of metals* or their oxides are characterized by electrochemical activity as well as a large surface. In biosensors and sensors, nanoparticles of noble (Au, Ag, Pt, Pd) and transition metals (Cu, Ni, Co., Fe, etc.) are actively used [209,210]. They act as signal-forming labels [211,212], carriers for the receptor layer, and transducer components [213,214,215]. The use of metal nanoparticles makes it possible to improve the analytical characteristics of the sensors due to increasing the conductivity of the transducer, the possibility of amplifying the analytical signal, and the covalently oriented immobilization of the receptor layer (usually according to the thiol-disulfide reaction). Therefore, gold and silver nanoparticles have found wide application as signal-forming labels in immunosensors for the determination of bacterial and viral agents [216]. The authors of [217] developed a device containing two separate graphite working electrodes, which was used to detect human IgG and goat IgG using gold nanoparticles as signal-forming labels. A similar device was used for the quantitative determination of a carcinoembryonic antigen (CEA) and α-fetoprotein (AFP) using antibodies labeled with gold nanoparticles [218]. In the works [219,220,221], previously thiolated gold and silver nanoparticles were used to covalently immobilize antibodies on the transducer surface. Ag-SiO_2_ nanoparticles were applied as a carrier for a fluorescent signal-forming label in sensor construction for the detection of *Escherichia coli* [222]. An original approach to the determination of *Staphylococcal enterotoxin B* has been proposed [223]. “ZnS-quantum dots” were used as a signal-forming label. The developed method is characterized by C_lim_ 0.2 ng/mL for fluorescent detection and 0.24 ng/mL for electrochemical detection.

The use of *magnetic nanomaterials (MNM)* allows for including stages of magnetic separation and magnetic concentrating in the analysis procedure. Concentrating in a magnetic field is an express and simple method, which does not require the use of expensive and large-sized equipment.

Paramagnetics, such as iron oxides [224,225], ferrites of manganese, cobalt, and nickel, and their composites: metal–metal, metal oxide–metal, metal–(bio) polymer, etc. (for example, FePt, Fe_3_O_4_-Ag, Fe_3_O_4_-Au, and CdS-FePt), have been widely used in sensory devices [226,227,228,229,230]. Nanomaterials are functionalized to increase the efficiency of their binding to biomolecules [231,232,233,234] and to use the coating to form an analytical signal [235,236].

The authors in [237] developed an immunosensor for CEA detection on the base of magnetic nanoparticles (MNPs). Primary antibody conjugated with magnetic iron-oxide nanoparticles that were coated with silica capture the target antigen. After that, a secondary antibody modified with SiO_2_ nanoparticles was added. SiO_2_ provided more binding sites for the signal-forming label’s (cyanine fluorophore) immobilization. It resulted in a 6-fold increase in fluorescence and thus heightened sensitivity. Electrochemical immunosensors for the quantitative determination of *Escherichia coli* ATCC 25992 and *Staphylococcus aureus* B-1266 were proposed [238,239]. Fe_3_O_4_ MNPs, coated with chitosan or 3-aminopropyltriethoxysilane, have been used as a direct signal-forming label. The linear range for both immunosensors is estimated to be 10–10^5^ CFU/cm^3^. The detection limit for *Escherichia coli* ATCC 25992 determination in water samples is 9.3 CFU/cm^3^. For *Staphylococcus aureus* determination, C_lim_ = 8.7 CFU/cm^3^. An electrochemical immunosensor based on an Fe_3_O_4_-SiO_2_ nanocomposite as a direct signal-forming label for measles virus antigen detection is described in [240]. The detection limit is equal to 1.87 × 10^−5^ mg/cm^3^. Metal–polymer nanocomposites based on Fe_3_O_4_ were used as signal-forming labels for the quantitative determination of *Escherichia coli* ATCC 25922 in the environment [235]. Amorphous ferrocene-modified silica was used as an electrochemically active polymer coating. The detection limit for the developed immunosensors is 12 CFU/cm^3^ for *E. coli* detection [241].

Nevertheless, the problem of producing nanostructures containing sensors arises due to the instability of suspensions of nanoparticles. Such disadvantage is eliminated mainly by a preliminary modification of the surface of the nanoparticles or substrate with the aim to increase their binding to the surface or to cover them by polymer.

## 4. Sensors Design

### 4.1. Chips, Microfluidic Systems, and Lab-On-The-Chips

A chip is a microelectronic measuring device used both in laboratory practice and in the home clinic [242,243]. The sensitive element of the chip is a sensor. The design features of chips are determined by the nature of the response and the required analytical characteristics. Chips, as a rule, are built into microfluidic systems. Microfluidic systems are adopted to work with extremely small flows of liquids (less than 10^−6^ L). The systems differ in the way they organize the flow. *Continuous Flow Based Microfluidics* provide a continuous flow of reagents through microchannels [244,245]. They are suitable for solving analytical problems that do not require complex liquid manipulations (flow chemical monocomponent analysis). In *Droplet Microfluidics Based Chips* or *Micro-Electrode-Dot-Array* systems, the reagents pass as droplets suspended in the carrier phase [246,247]. Such systems are more labile, universal, and suitable for multiplex analysis of complex samples.

Of great interest today are analytical *lab-on-the-chip* systems, including a sensor integrated into a chip, microfluidic transport of the reagents, and a portable detector. Having regard to the many advantages of *lab-on-a-chip* technologies, including multiplex analysis, the small size of the device, the low reagent consumption, and easy integration, such systems are widely used in drug screening, cell engineering, and medical diagnostics [248,249]. Lab-on-the-chips are easy to integrate into smartphones. For example, a lab-on-the-chip for the detection of three important mycotoxins (ochratoxin A (OTA), aflatoxin B1 (AFB1), and deoxynivalenol (DON)) in spiked buffer was developed [250]. A colorimetric signal for performing a multiplexed semi-quantitative direct competitive ELISA has been detected using a smartphone camera. Horseradish peroxidase was used as an enzymatic label. The reagents have been flown though the reaction chambers using pumps. Detection limits of <40, 0.1–0.2, and <10 ng/mL were obtained for OTA, AFB1, and DON, respectively. A disposable paper–plastic hybrid microfluidic lab-on-a-chip has been developed for the colorimetric measurement of some components in urine [251]. The developed device is incorporated into a paper-based conventional reagent test strip inside a plastic-based microchannel. For the colorimetric reaction of glucose, protein, pH, and red blood cells, the device uses a small volume (40 μL) of urine and a finger-actuating micropump. The lab-on-the-chip is controlled by a smartphone-based optical platform using the “UrineAnalysis” Android app.

The use of synthetic receptors in designs of lab-on-the-chips for bioanalytes detection allows for the creation of sensitive and stable devices for in vitro and in vivo medical diagnostics.

### 4.2. Wearable and Written Sensors

**Figure 3:**
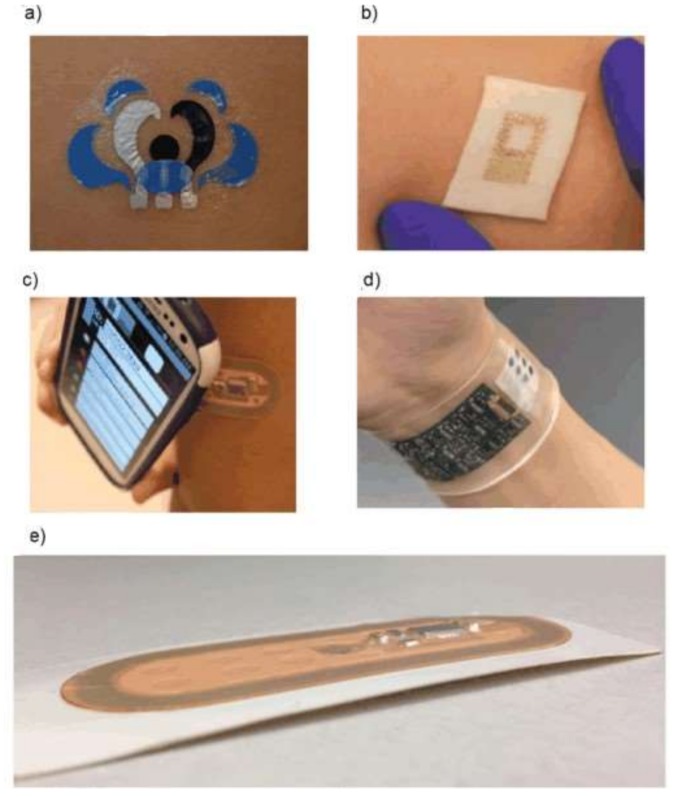
Three types of wearable sensors: tattoo (**a**,**b**), patch (**c**), and band (**d**,**e**) [255].

Wearable sensors are presented by soft, flexible, and stretchable electronic devices intended for the monitoring of clinically important indices of human health [252,253,254]. Chemical wearable sensors, preferably with an electrochemical detection of the response, are used for the determination of individual chemical substances and groups of the components (biomarkers). Designs of electrochemical wearable devices have been distinguished, namely, tattoo, patch, and band [255]. Tattoo sensors are manufactured using specially engineered stress-enduring inks and biocompatible polymers. These are the most ergonomic and convenient-to-use sensors. Patch sensors made on a textile basis are more durable, have a more developed surface, and, therefore, have more options for immobilizing the receptor layer and integrating the accompanying electronics. Tattoo and patch sensors are low-cost and not intended for re-use. Band sensors based on silicone are the most durable and reliable of all the above devices, and there is the possibility of replacement/regeneration of the sensor layer as well as multiplex detection of analytes. Figure 3 shows real examples of such sensors.

The authors of [256] have developed an enzyme biosensor for the determination of lactate in sweat using a flexible printed temporary-transfer tattoo. The receptor layer based on lactate oxidase is coated with chitosan. Potentiometric sensor patches intended for the detection of calcium, ammonium, and sodium ions are presented in the paper [257,258,259]. It was shown that such skin sensors are resistant to mechanical deformations and demonstrate practically the Nernstian response in a wide range of concentrations.

An integrated multi-analyte potentiometric-amperometric sensor wristband platform has been developed [260]. This new platform has combined a plastic-based sensor array with silicon-integrated circuits consolidated on a flexible circuit board to improve signal conditioning, processing, and transmission. In addition to monitoring the general profile of human sweat, it is possible to independently determine glucose, lactate, potassium, and sodium in exercise-induced sweat.

Recent advances in screen-printed film and nanotechnologies allow for the integration of thin polymeric, metal, carbon, and composite materials into electronic soft and stretchable devices [261,262].

An interesting strategy is the use of “island-bridge” layers, where current-conducting tracers are intertwined with functionalized “islands” or “serpentine-shaped” structures consisting of periodic arcs and straight segments, which have been adopted to connect rigid areas on top of soft elastomers [263,264,265]. Sometimes, wearable sensors have a core–shell structure [266,267,268]. The core is an ultra-thin sensor module packaged in a soft shell based on textiles or silicone. Such a design allows for the provision of the most durable contact with the skin and resistance to wetting.

Amperometry, potentiometry, and impedimetry are used as measuring methods. Principles of field-effect transistors are used as well. An organic field-effect-transistor-type (OFET) sensor based on nanoparticle-decorated reduced graphene oxide (Pt_rGO) for the determination of dopamine has been proposed by the authors of [269]. A conducting-polymer source–drain electrode modified with graphene acts as a transducer. The OFET sensor is flexible and shows a high sensitivity to remarkably low dopamine concentrations (10^−16^ M) and selectivity to relatively interfering molecules. An ultrasensitive impedimetric wearable immunosensor for the determination of cortisol has been proposed [270]. Antibodies are immobilized on thin films of zinc oxide, and the analytical signal is formed as a result of a change in the thickness of the electrical double layer due to antibody–hormone interactions at diagnostically relevant concentrations. The detection limit is 1 ng/mL and the linear range is 10–200 ng/mL.

**Figure 4:**
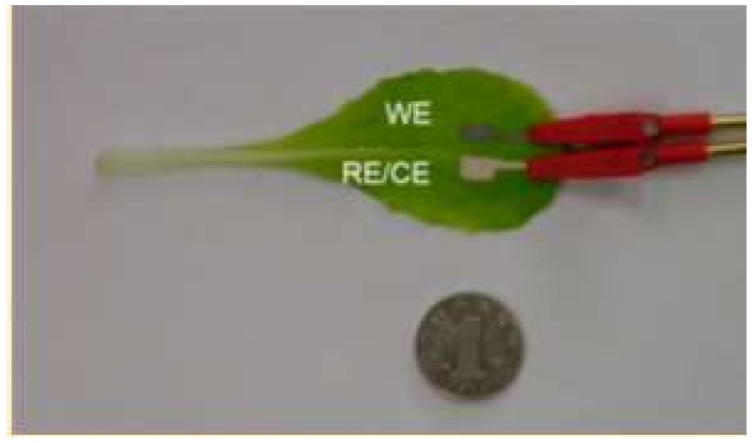
An electrochemical written sensor for the determination of methyl parathion and nitrite in food [271].

A written electrochemical sensor for the determination of methyl parathion and nitrite in food in situ has been proposed [271]. Written electrodes were made of a mixture of chitosan, graphite, and silver powders. The porous structure of the sensors makes it possible to perform an analysis without preliminary extraction of an analyte. The written sensor (Figure 4) was tested on model systems and real objects (Fuji apples, Chinese onions, and cabbage). The detection limit is 15 ng and 18.4 μg for methyl parathion and nitrite, respectively.

**Figure 5:**
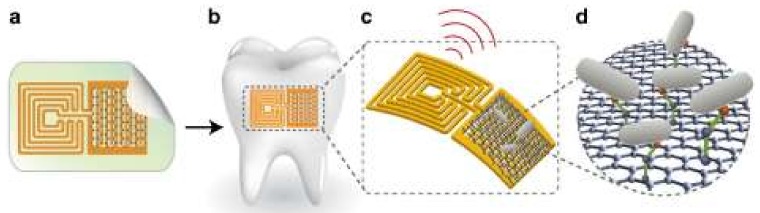
Design of the graphene-based hybrid nanosensor: (**a**) printing of graphene onto a silk film; (**b**) biotransfer of the nanosensor onto a tooth; (**c**) schematic of the sensing element; (**d**) binding of the bacteria by a self-assembling antimicrobial peptide [272].

A biotransferrable graphene wireless nanosensor for bacteria detection in saliva was developed [272]. Figure 5 shows the design of the sensor.

Firstly (Figure 5a), a graphene-based sensing element with a wireless readout coil was generated on silk fibroin. Secondly, the graphene-based transducer was biotransferred onto a tooth surface followed by dissolution of the supporting silk film (Figure 5b). Next, the specificity of detection was provided by the use of self-assembling antimicrobial peptides (odorranin-HP) onto the graphene monolayer. When recognition and binding of the target bacteria (*Helicobacter pylori*) are realized, electroconductivity of the graphene film is modulated and wirelessly monitored using an inductively coupled radio frequency reader device (Figure 5c,d). The developed hybrid nanosensor was demonstrated to have a low detection limit (100 CFU/mL), battery-free operation, and a remote wireless sensing capability.

**Figure 6:**
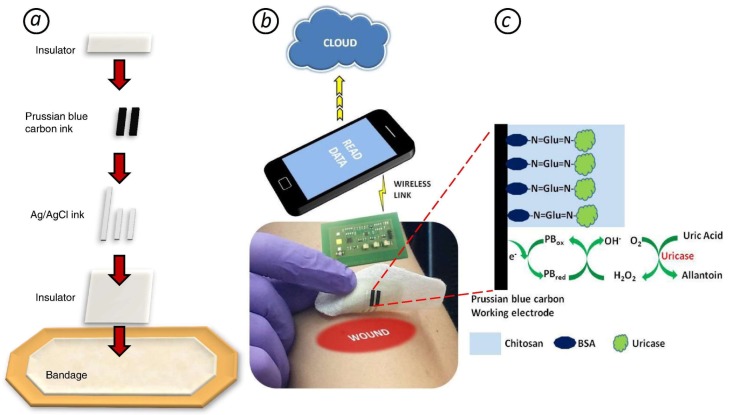
(***a***) Screen printing the smart bandage. (***b***) Wearable potentiostat determines uric acid (UA) concentration and wirelessly communicates with a computer or smartphone. (***c***) The principle of UA amperometric detection (BSA - Bovine serum albumin) [273].

The authors of [273] developed a smart bandage for uric acid (UA) detection. UA level in wound exudate is highly correlated with wound severity and indicates the bacterial infection of it (a significant decrease because of catabolysis by microbial uricase). The device is based on a urease/prussian blue amperometric screen-printed biosensor and a custom-designed wearable potentiostat, which allowed for on-demand wireless data transfer to a computer/smartphone using radio frequency identification or near-field communication (Figure 6). The authors demonstrated high sensitivity, selectivity, operational stability, and mechanical robustness for the developed sensor. The use of the smart bandage allows for optimizing the process of wound healing and reducing a patient’s stress and pain.

Prospects for the development of wearable and written sensors are extensive, and the scope of their potential application is enormous. Miniature and wireless sensors allow for constant monitoring of a patient’s health both at the bedside and far beyond the hospital. At the same time, due to modern built-in data exchange systems, a doctor’s recommendations can be received at a distance. Thus, the development of wearable and written sensors is an important step towards personalized medicine.

However, some problems exist that should be solved before the sensors of this kind find application in practice. They are the low reliability of such modern devices and the dependence of measurement results on the efficiency of biological fluids transport (for example, sweat) and analytes to the surface of the sensor. The authors also point out problems with the selectivity of the analysis and the possibility of the sensors to be re-used [274,275]. Data interpretation as a rule is ambiguous. It is necessary to establish if a correlation between measurement parameters that are characteristic for the skin and blood is observed.

### 4.3. Powering of Sensors

**Figure 7:**
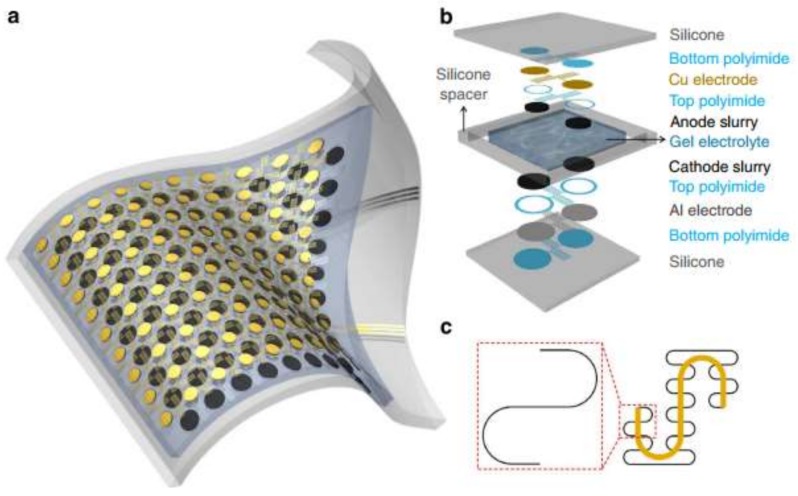
Battery layout and design. (**a**) Scheme of a battery’s construction; (**b**) Illustration of the various layers in the battery’s structure; (**c**) ‘Self-similar’ serpentine geometries scheme (black: 1st level serpentine; yellow: 2nd level serpentine) [284].

Processes occurring in the immediate environment, directly on the body of the carrier, can serve as the source of energy for a sensor. A strategy for supplying power to portable electronics from the energy of human body movement using a triboelectric nanogenerator (TENG) has been proposed [276,277,278]. Devices operating from solar panel, vibratory, radio frequency, electromagnetic, thermal, chemical-to-electric, and biofuel cells are described. However, the complexity of the power suppliers’ designs and the need to take into account a large number of factors (the location of the device, the degree of physical activity of the user, the duration of daylight, etc.) to ensure the reliable operation of the sensor, especially in the monitoring mode, does not yet allow them to be widely implemented. Today, flexible and stretchable batteries are also used as a source of energy [279,280,281]. Such batteries should be thin, but at the same time, they should have a sufficient charge capacity. Technologies of manufacturing thin-layer printed batteries make it possible to successfully solve these problems [282,283]. For example, the authors in [284] developed a stretchable lithium ion battery on a base of thin, low-modulus silicone elastomers segmented with metal electrodes and electrolyte layers (Figure 7). Self-similar serpentine interconnecting geometries allowed the authors to achieve extremely high stretchability while maintaining capacity densities of 1.1 mAh/cm^2^.

**Figure 8:**
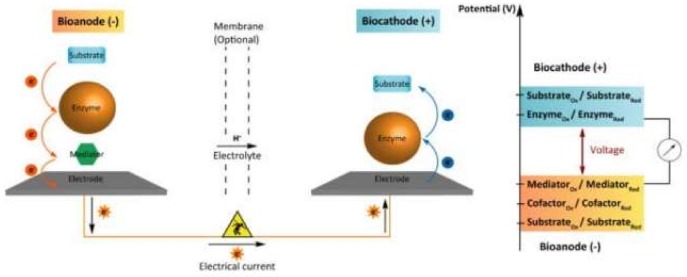
Schematic of an enzymatic biofuel cell involving a mediated bioanode and a direct electron transfer-based biocatode [285].

In the last few years, researchers have been interested in self-powered sensors based on the biofuel cells principle [285]. The transformation of biocatalytic reaction energy into electrical energy makes it possible to create miniature, portable, and energetically autonomous sensors. Enzyme, organelle, and microbial biofuel cells and mediated electron transfer can be realized (see Figure 8).

The mediator transfers electrons from the enzyme to the anode. At the cathode, a direct electron transfer from the electrode to the enzyme occurs. Small molecules or redox polymers are used as mediators of electron transfer. For example, a self-powered biosensor system for the determination of cholesterol is described in [286]. At the anode, enzymatic oxidation of cholesterol occurs. The hydrogen peroxide formed during the reaction is catalytically reduced on the cathode modified with Prussian blue.

One sensitive element of organelle-based biofuel sensors are cellular organelles: membranes, mitochondria, etc. The principles of forming the response of organelle-based biofuel sensors are similar to those for enzymatic biofuel cells. However, due to the habitual microcellular environment, organelle-based sensors are more stable but significantly inferior to enzymatic sensors for selectivity. In addition, organelles are extremely sensitive to toxins. In [287], a sensor for cyanide detection based on the toxic effect of organelles on the mitochondria of protozoa microorganisms is described. A similar effect was used in [288] for the development of sensors sensitive to herbicides. The main component of the receptor layer in this case was thylakoid membranes of plants.

Microbial biofuel sensors use microbial fuel cells (MFCs) as a receptor layer. MFCs perform the function of the biocatalyst of substrate oxidation and carry out the transfer of electrons to the anode by intracellular mechanisms. Then, electrons move to the cathode along the external circuit, which generates power [289]. There are turn-on and turn-off microbial biofuel sensors. Thus, in the pioneering work [290] *Clostridium butyricum* are used in the design of a turn-on self-powered biosensor for the monitoring of biological oxygen demand. The inhibitory effect of organic toxicants on microorganisms underlies turn-off microbial biofuel sensors [291]. Such self-powered biosensors have been published in [292,293,294,295,296]. For example, the authors of [297] have developed a single-chamber MFC that could be operated in flow-mode for bacteriological wastewater analysis.

The main problem of self-powered biosensors is the low stability of the enzymes. The use of synthetic receptors will improve the analytical characteristics and extend the shelf life of these sensors.

## 5. Applications of Sensors

Biosensors are developed for a range of applications. Nevertheless, only some of them have found real practical application today [298]. Examples are “Contour” for glucose determination, “OneTouch” for glucose and cholesterol determination [299,300], test strips sensitive to hCG [301], a lactose biosensor [302,303], and portable systems for enzyme immunoassay and polymerase chain reaction analysis [304,305,306,307]. Despite a large number of publications, the potential field of application for biosensors is huge, and many existing developments are far from where we should expect them to be, usually due to their low reliability and the complexity of their design. In our opinion, sensors with synthetic receptors (MIPs, aptamers, biomimetics) have great prospects for commercial implementation due to their stability and sensitivity.

### 5.1. Medical Diagnostics

Medical diagnostics, of course, is the main field of practical application for sensors. Simplicity of use, short response time, and high sensitivity and selectivity make them indispensable for onsite and in situ application, for example, in the home clinic. Special attention is paid to the diagnostics of such socially significant diseases as diabetes, cancer, and infectious and cardiovascular diseases. A continuous search for new biomarkers and the development of methods for their detection is in progress.

Oncological diseases occur in a healthy organism suddenly and develop very intensively, so early diagnosis in this case plays a key role. The level of oncomarkers in a patient’s blood at the initial stages of the disease does not exceed a few pM, so not many methods and sensors allow for their detection. Aptasensors have been successfully applied in this field. For example, a voltammetric sensor [308] based on a methylene blue-labeled aptamer for determination of the cancer marker vascular endothelial growth factor (VEGF) has been described. The receptor layer is immobilized on the surface of a gold-plated screen-printed electrode due to thiol-disulfide interaction. The detection limit is 190 ng/L. An aptasensor [309] sensitive to platelet-derived growth factor BB (PDGF-BB) has been developed. The authors have used the strategy of amplifying the response of hydroxyapatite nanoparticles (HAP-NPs), which were a support for the deposition of the aptamer. Because of the aptamer’s immobilization on the electrode surface, a redox-active molybdophosphate precipitate was formed. The developed amperometric aptasensor is characterized by high sensitivity (C_lim_ = 50 fg/mL) and a wide linear range (0.1 pg/mL–10 ng/mL).

No less important is the detection of cardiomarkers. An aptasensor for the determination of myoglobin has been proposed. A myoglobin-specific aptamer was immobilized on the surface of a printed electrode and modified with graphene oxide and carbon nanotubes, which were used in this device [310]. The sensor provides a low detection limit (34 ng/L) and a linearity range of (1–4000 ng/mL) was observed.

The risk of a cardiovascular disease is directly related to the cholesterol concentration in the blood. An enzyme-free sensor sensitive to cholesterol has been proposed [311]. Nanohybrid silica-chitosan oligosaccharide lactate particles were coated with a curcumin layer. They fluoresce in the presence of cholesterol interacting with it electrostatically. A non-enzymatic method for cholesterol determination based on cobalt chloride has been proposed [312]. The analytical signal is an increase in the oxidation peak of cobalt chloride in an aprotic medium in the presence of cholesterol. The detection limit of the method is 2 μM and its linear range is 25–200 μM. The results obtained are lower than the cholesterol content in venous blood. The difference can be due to the fact that capillary blood was analyzed. This phenomenon should be thoroughly investigated before using the sensor.

Despite the commercial success of classical glucose meters, non-enzymatic sensors for the rapid determination of blood glucose are being actively developed today. A sensor based on three-dimensional porous Cu/Cu_2_O films decorated with Pd nanoparticles acting as an electrocatalyst was proposed [313]. Its high detection sensitivity (C_lim_ = 1.157 mA cm^−2^ mM^−1^) is caused by the porous structure of the modifier and the high catalytic activity of Pd nanoparticles. A catalyst for the non-enzymatic determination of glucose based on copper nanoparticles electrodeposited on the surface of a Ni-based metal-organic framework derivate is described in [314]. The sensor has a low detection limit of 66.67 nM and a wide linear range of 0.20 to 2.72 mM. It is highly stable and suitable for re-use. A poly(m-phenylenediamine)/sliver (PmPD/Ag) composite electrode was used [315] for non-enzymatic glucose detection. An amperometric sensor was formed by the in situ synthesis of Ag particles on the surface of the polymer matrix. The sensor is sensitive (C_lim_ = 7.2 μM), fast (the response time is 3 s), and stable for 30 days.

In the case of bacterial and viral agents diagnostics, more stable and sensitive sensors based on synthetic receptors (aptamers and MIPs) have replaced the traditional biosensors (immunosensors, DNA sensors). An impedimetric sensor for *Staphylococcus epidermidis* detection has been developed [316]. The selectivity of the sensor is caused by a cell-imprinted polymer based on 3-aminophenylboronic acid. Its linear range is 10^3^–10^7^ CFU/mL. Even better analytical characteristics were achieved in [317], where a very cost-effective, fast, sensitive, and specific electrochemical sensor for the detection of *Escherichia coli* modified with an imprinted polymer is described. The sensor is based on Ag-ZnO bimetallic nanoparticles and a graphene oxide nanocomposite, which serve as a platform for the imprinting of bacteria. The signal of the K_3_[Fe(CN)_6_]/K_4_[Fe(CN)_6_] mediator system was detected voltammetrically using a glassy carbon electrode. The detection limit of the sensor is less than 10 CFU/mL and the linear range is 10–10^5^ CFU/mL. A fluorescent aptasensor for the determination of H5N1 influenza virus has been proposed [318]. Ag-SiO_2_ nanoparticles were used as a metal-enhanced fluorescence sensing platform, and thiazole orange served as a signal-forming label. The duration of analysis did not exceed 30 min, and the detection limit of H5N1 in serum is 3.5 ng/mL.

### 5.2. Environmental and Food Analysis

Сontaminants detection (pesticides, toxins, antibiotics, heavy metals, etc.) in food and the environment is a very important task. A wide application in environmental and food analysis is found for enzymatic sensors, in which an analyte is an inhibitor of the biocatalytic activity and immunosensors. The use of sensors allows for *in situ* and onsite determination, eliminating the sample preparation stage. This greatly simplifies and speeds up the analysis. In addition, there is the possibility of a multiplex analysis. Many papers on sensors for environmental and food analysis are published today [319,320,321]. Below are some examples of contaminants determination in environmental objects and food.

An electrochemiluminescent aptasensor for the detection of malachite green and chloramphenicol has been proposed [322]. Malachite green is potential carcinogenic and mutagenic agent, while chloramphenicol causes gray baby syndrome, leukemia, and aplastic anemia. CdS quantum dots and luminol-gold nanoparticles decorated on a homemade screen-printed carbon electrode were used as signal-forming labels. The detection limits for malachite green and chloramphenicol were 0.03 nM and 0.07 nM, respectively. Chen et al. [323] have developed a method for the detection of chloramphenicol and polychlorinated biphenyl-72 based on the displacement of a nanotracer (an aptamer-labeled magnetic dendritic probe) by the analytes. Thiolated aptamers were assembled on Fe_3_O_4_@Au nanoparticles which were used for concentrating the analyte. At the second stage, nanotracers having in their structure hybridized complementary strands (cDNA1 or cDNA2) and quantum dots (CdS or PbS) were formed. Next, a hybridization reaction between aptamers and cDNAs produces composite magnetic probes. Once the analytes are recognized, the nanotracers are released and detected simultaneously by voltammetry of the metal ions Cd(II) and Pb(II).

At present, many sensors for the determination of mycotoxins in food and environmental objects have been developed. Thus, nanostructured polymer membranes synthesized in situ have become the basis of a fluorescent sensor for the selective determination of aflatoxin B1 [324]. The detection limit of the sensor is 14 ng/mL and its linear range is 20–160 ng/mL. A label-free impedimetric aptasensor for ochratoxin A detection based on immobilized thiol-modified aptamers on Ag nanoparticles, which were decorated on the gold surface, is proposed in [325]. The analytical signal is detected in the presence of a ferricyanide redox mediator. The sensor is tested on model solutions and real beer samples. The detection limit of the sensor is 0.02 nM.

## 6. Trends and Prospects

Bio- and chemical sensors play an essential role in the implementation of innovative and integrated techniques for the monitoring of health, applications in food analysis and quality control, and environmental applications, for example, the assessment and monitoring of the quality of water resources, as an alternative to the classical chemical analytical methods. Vikas, Anjum, and C S Pundir in the review published in 2007 [326] wrote: “Biosensors offer considerable promises for attaining analytic information in a faster, simpler, and cheaper manner compared to conventional assays. The biosensing approach is rapidly advancing, and applications ranging from metabolite, biological/chemical warfare agent, food pathogens, and adulterant detection to genetic screening and programmed drug delivery have been demonstrated. Innovative efforts, coupling micromachining and nanofabrication may lead to even more powerful devices that would accelerate the realization of large-scale and routine screening. With gradual increase in commercialization, a wide range of new biosensors are thus expected to reach the market in the coming years”. Their prediction turned out to be correct.

A great majority of the biosensors described earlier and used today are based on an application of enzymes as the recognition component. Their advantage is the high selectivity. Their disadvantages are instability, cost, and the need in some cases to use them in combination with a mediator system. These disadvantages are why the tendency to get rid of enzymes can be observed. Researchers are focused on the search for and synthesis of compounds that exhibit the properties of receptors (sensing elements of sensors), catalysts, and compounds that provide selectivity of detection and signal-forming labels. Nanostructures, organic and inorganic compounds containing transition metals, electrochemically active compounds, synthetic analogues of enzymes, and MIPs are used as such materials as an alternative to enzymes. Moreover, magnetic materials are used as carriers accelerating the delivery of a signal-forming complex to the electrode surface. Replacement of enzymes with alternative materials makes it possible to create a fundamentally new generation of non-enzymatic biological and chemical sensors that are not inferior to the classical ones in terms of sensitivity, selectivity, and accuracy of determination of target analytes, but that are much more stable during storage, are commercially available, and are versatile in use.

A significant role in the development of sensorics is played by new approaches to the design of sensors, such as a lab-on-a-chip, printed (written), and wearable sensors. Using the principles of fuel cells can reduce the energy consumption of measuring systems and provide the possibility to create miniature versions of them. The electrochemical method of detection remains the preferred one. The results of these studies will serve as a basis for creating devices that are needed not only in laboratory clinical trials, but also in point-of-care diagnostics, the home clinic, and telemedicine.
